# Loss of Pten synergizes with c-Met to promote hepatocellular carcinoma development via mTORC2 pathway

**DOI:** 10.1038/emm.2017.158

**Published:** 2018-01-05

**Authors:** Zhong Xu, Junjie Hu, Hui Cao, Maria G Pilo, Antonio Cigliano, Zixuan Shao, Meng Xu, Silvia Ribback, Frank Dombrowski, Diego F Calvisi, Xin Chen

**Affiliations:** 1 Department of Gastroenterology, Guizhou Provincial People’s Hospital, The Affiliated People’s Hospital of Guizhou Medical University, Guiyang, PR China; 2 Department of Bioengineering and Therapeutic Sciences and Liver Center, University of California, San Francisco, CA, USA; 3 School of Pharmacy, Hubei University of Chinese Medicine, Wuhan, PR China; 4 Department of Oncology, Guizhou Provincial People’s Hospital, Guiyang, PR China; 5 Institute of Pathology, University of Greifswald, Greifswald, Germany; 6 Lowell High School, San Francisco, CA, USA; 7 Department of Hepatobiliary Surgery, The First Affiliated Hospital of Xi'an Jiaotong University, Xi’an, PR China

## Abstract

Hepatocellular carcinoma (HCC) is a deadly malignancy with limited treatment options. Activation of the AKT/mTOR cascade is one of the most frequent events along hepatocarcinogenesis. mTOR is a serine/threonine kinase and presents in two distinct complexes: mTORC1 and mTORC2. While mTORC1 has been extensively studied in HCC, the functional contribution of mTORC2 during hepatocarcinogenesis has not been well characterized, especially *in vivo*. Pten expression is one of the major mechanisms leading to the aberrant activation of the AKT/mTOR signaling. Here, we show that concomitant downregulation of Pten and upregulation of c-Met occurs in a subset of human HCC, mainly characterized by poor prognosis. Using CRISPR-based gene editing in combination with hydrodynamic injection, Pten was deleted in a subset of mouse hepatocytes (sgPten). We found that loss of Pten synergizes with overexpression of c-Met to promote HCC development in mice (sgPten/c-Met). At the molecular level, sgPten/c-Met liver tumor tissues display increased AKT and mTOR signaling. Using *Rictor* conditional knockout mice, we demonstrate that sgPten/c-Met-driven HCC development strictly depends on an intact mTORC2 complex. Our findings therefore support the critical role of mTORC2 in hepatocarcinogenesis. sgPten/c-Met mouse model represents a novel valuable system that can be used for the development of targeted therapy against this deadly malignancy.

## Introduction

Hepatocellular carcinoma (HCC) is one of the major causes of morbidity and mortality worldwide, especially in less developed countries.^1^ Currently, with the exception of the multikinase inhibitors Sorafenib and Regorafenib, the therapy options for patients with unresectable or metastatic HCC are very limited. However, patients with advanced HCC only experience ~3 months of benefits from Sorafenib or Regorafenib treatment.^2, 3^ Consequently, it is imperative to elucidate the molecular pathogenesis of HCC in order to develop innovative therapies against this malignancy.

It is well established that the Phosphoinositide-3-Kinase (PI3K)/v-AKT Murine Thymoma Viral Oncogene Homolog 1 (AKT) pathway is frequently dysregulated in cancer.^4, 5, 6^ By activation of AKT and other downstream effectors, the PI3K pathway regulates a broad spectrum of processes essential for cancer, including cell survival, proliferation, growth, metabolism and angiogenesis.^6, 7, 8^ The PI3K pathway can be activated by genetic alterations in PIK3CA, TSC1/2, LKB1 and Pten, or by the activation of upstream inducers such as IGF and HGF/c-Met signaling. This complex signaling network has been shown to play a critical role in hepatocarcinogenesis.^9, 10, 11^ In normal tissues, the PI3K/AKT pathway is negatively regulated by the tumor suppressor phosphatase and tensin homolog (Pten).^8^ Expression of Pten is reduced in about half of all HCC tumors, leading to constitutive activation of the PI3K/AKT pathway.^12, 13^

The c-Met proto-oncogene encodes the receptor for hepatocyte growth factor (HGF). HGF-induced c-Met activation drives an intricate cascade of molecular events, involving multiple downstream targets, such as the mitogen-activated protein kinase (MAPK) and PI3K pathways. The c-Met signaling has been shown to promote tumor invasion and metastasis by sustaining cell proliferation, survival, migration and angiogenesis.^14, 15, 16^ c-Met is often overexpressed in human HCC samples and considered to be a therapeutic target in this disease.^15, 17, 18^

The serine/threonine kinase mTOR is one of the major downstream effectors of PI3K signaling. mTOR acts as part of two distinct multiprotein complexes, mTOR complex 1 (mTORC1) and mTOR complex 2 (mTORC2).^19, 20^ mTORC1 functions via regulating cellular growth and metabolism, and it is highly sensitive to Rapamycin. The major downstream targets of mTORC1 include p70 ribosomal S6 kinase (p70S6K) and eukaryotic translation initiation factor 4E-binding protein 1 (4E-BP1). p70S6K phosphorylates PRS6, leading to increased glycolysis and lipogenesis. 4E-BP1 functions together with eukaryotic translation initiation factor 4E (eIF4E) to regulate CAP-dependent translation. Unlike mTORC1, how mTORC2 is regulated and its functional contribution to tumorigenesis remain poorly understood.^4, 20^ AGC kinases, which include AKT, SGK and PKC-α, are considered to be the major substrates of mTORC2, and in turn regulate cell cycle progression, cell survival, and metabolism. In human HCC, AKT has been found to be activated in ~50% of tumor specimens, and is associated with aggressive tumor growth and poor prognosis.^21, 22^ Our recent and other studies demonstrate that an intact mTORC2 is required for the activation of AKT *in vitro* and *in vivo*.^13, 23^ Furthermore, chromosomal gain of Rictor, the unique and essential subunit of mTORC2, is found in ~25% of human HCCs.^13^ These data indicate the activation mTORC2 in HCCs. However, the precise mechanisms whereby mTORC2 modulates hepatocarcinogenesis remains poorly understood.

We recently showed the oncogenic cooperation between PI3K/AKT and c-Met pathways along liver carcinogenesis.^24^ As loss of Pten expression is a major mechanism leading to activated PI3K/AKT signaling, we investigated whether ablation of *Pten* synergizes with c-Met to promote HCC development *in vivo*. We test the hypothesis by using CRISPR-based technology to delete Pten (sgPten) while co-expressing c-Met using sleeping beauty transposon system and hydrodynamic transfection (sgPten/c-Met). Using sgPten/c-Met induced HCC as preclinical model and conditional *Rictor* KO mice, we demonstrated the critical role of mTORC2 in hepatocarcinogenesis.

## Materials and methods

### Human liver tissue specimens

A collection of formalin-fixed, paraffin-embedded HCC samples was used in the present study. Fifty frozen HCC and corresponding non-tumorous surrounding livers from the same collection were used. Tumors were divided in HCC with shorter survival/poorer prognosis (HCCP; *n*=25) and longer survival/better prognosis (HCCB; *n*=25), characterized by <3 and >3 years’ survival following partial liver resection, respectively. The clinicopathological features of liver cancer patients are summarized in Supplementary Table 1. HCC specimens were collected at the Medical University of Greifswald (Greifswald, Germany). Institutional Review Board approval was obtained at the local Ethical Committee of the Medical University of Greifswald. Informed consent was obtained from all individuals.

### Constructs and reagents

The constructs used for mouse injection, including PX330-sgPten, pT3-EF1α-c-met, pT3-EF1α, pT3-EF1α-Cre and pCMV/sleeping beauty (SB) transposase, have been described previously.^24, 25, 26, 27, 28^ All the plasmids were purified using the Endotoxin-free Maxi prep kit (Sigma-Aldrich, St Louis, MO, USA) for *in vivo* experiments.

### Hydrodynamic injection and mouse monitoring

Wild-type FVB/N mice were obtained from Charles River Laboratories (Wilmington, MA, USA) and the *Rictor*^*fl/fl*^ mice^29^ from the Jackson Laboratory (Sacramento, CA, USA). Hydrodynamic injection was performed as described previously.^30^ In brief, the plasmids encoding the genes of interest along with SB transposase in a ratio of 25:1 were diluted in 2 ml saline (0.9% NaCl), filtered through 0.22 μm filter, and injected into the lateral tail vein of the mice in 5–7 s. For the tumorigenesis models, 20 μg sgPten, 20 μg c-Met with 1.6 μg SB plasmid were delivered into FVB/N mouse liver (*n*=14). To determine the requirement of mTORC2 in sgPten/c-Met-driven liver carcinogenesis, high dose of Cre (60 μg) or pT3-EF1α (60 μg) was mixed with sgPten (20 μg), c-Met (20 μg) and SB (4 μg) and injected into *Rictor*^*fl/fl*^ mice (*n*=11 and 5, respectively). Mice were housed, fed and monitored in accordance with protocols approved by the Committee for Animal Research at the University of California, San Francisco.

### Immunohistochemical staining

Liver specimens were fixed in 4% paraformaldehyde and embedded in paraffin. Liver histopathologic analysis on mouse lesions was assessed by two experienced liver pathologists (SR and FD) in accordance with the criteria described in detail previously.^31^ Immunohistochemistry (IHC) was performed as previously described.^21, 24^ The primary antibodies against c-Met (Abcam, Cambridge, MA, USA; 1:100), p-AKT^S473^ (Cell Signaling Technology, Danvers, MA, USA; 1:100), Pten (Cell Signaling Technology; 1:100), fatty acid synthase (FASN; Cell Signaling Technology; 1:150), acetyl-CoA carboxylase (ACC; Cell Signaling Technology; 1:100), p-ERK (Cell Signaling Technology; 1:100), and Ki67 (Thermo Fisher Scientific, Waltham, MA, USA; 1:150) were used in the present investigation.

### Western blot analysis

Frozen mouse liver specimens were homogenized in Mammalian Protein Extraction Reagent (Thermo Scientific, Waltham, MA, USA) containing the Complete Protease Inhibitor Cocktail and sonicated. Protein concentrations were determined with the Bio-Rad Protein Assay Kit (Bio-Rad, Hercules, CA, USA) using bovine serum albumin as standard. Supernatant was boiled in Laemmli sample buffer for western blot analysis as previously described.^24^ Equal loading was assessed by GAPDH and/or β-actin. The antibodies used are as follows: Pten (Cell Signaling Technology; 1:1000), c-Met (Cell Signaling Technology; 1:1000), p-Met (Cell Signaling Technology; 1:1000), p-AKT^S473^ (Cell Signaling Technology; 1:1000), p-AKT^308^ (Cell Signaling Technology; 1:1000), total-AKT (Cell Signaling Technology; 1:1000), p-c-Raf^S259^ (Cell Signaling Technology; 1:1000), p-GSK3β (Cell Signaling Technology; 1:1000), p-PKC (Cell Signaling Technology; 1:1000), p-PRAS40 (Cell Signaling Technology; 1:1000), p-mTOR (Cell Signaling Technology; 1:1000), p-4EBP1 (Cell Signaling Technology; 1:1000), p-RPS6 (Cell Signaling Technology; 1:1000), p-ERK1/2 (Cell Signaling Technology; 1:1000), total-ERK1/2 (Cell Signaling Technology; 1:1000), HK1 (Cell Signaling Technology; 1:1000), HK2 (Cell Signaling Technology; 1:1000), PKM1 (Cell Signaling Technology; 1:1000), PKM2 (Cell Signaling Technology; 1:1000), GAPDH (EMD Millipore, Temecula, CA, USA; 1:10 000), β-Actin (Sigma-Aldrich; 1:4000).

### Statistical analysis

Differences between two groups were analyzed with unpaired t test using Prism 6 Software (GraphPad, San Diego, CA, USA). *P*–values <0.05 were considered as statistically significant.

## Results

### Expression of c-Met and Pten in human HCC specimens

**Figure 1:**
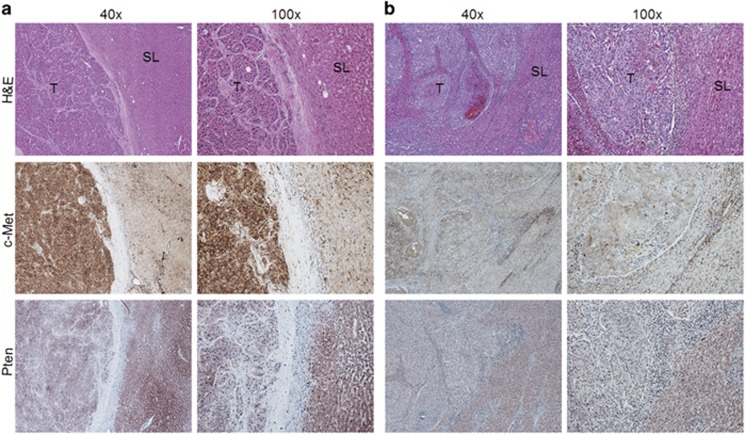
Expression patterns of c-Met and Pten in human hepatocellular carcinoma (HCC) as detected by immunohistochemistry. (**a**) HCC case showing upregulation of c-Met and downregulation of Pten in the tumor part (T), when compared with non-tumorous surrounding liver (SL). (**b**) HCC case showing equivalent levels of c-Met immunoreactivity in HCC and adjacent non-tumorous liver, while Pten immunolabeling is higher in the non-neoplastic compartments. Original magnifications: × 40 and × 100. H&E, hematoxylin and eosin staining.

**Figure 2:**
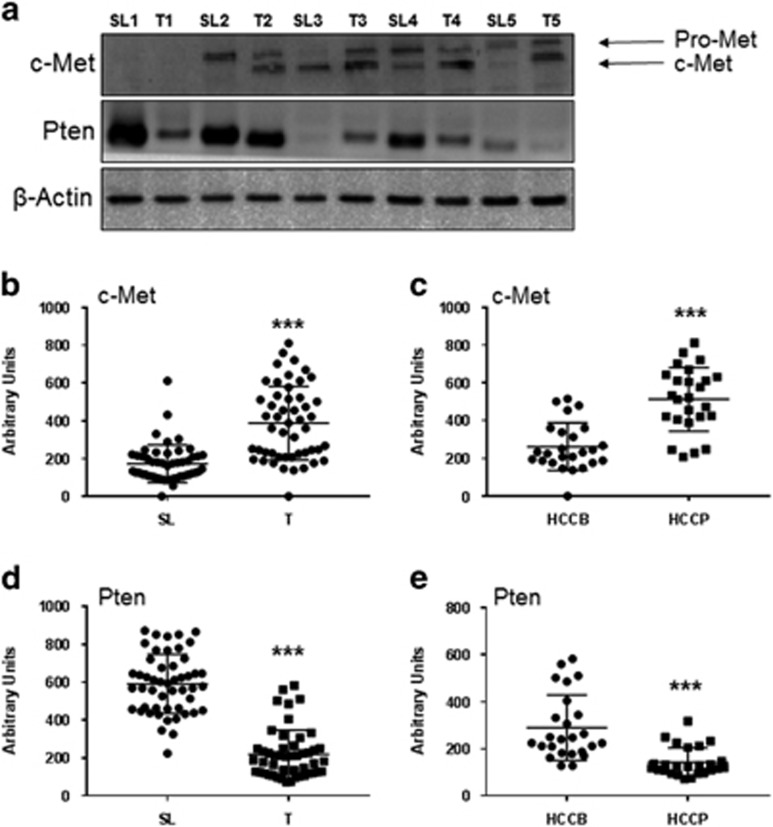
Protein expression patterns of c-Met and Pten in human hepatocellular carcinoma (HCC). (**a**) Representative immunoblotting of c-Met and Pten expression in five paired surrounding liver (SL) and human HCC (T) samples. β-Actin was used as loading control. (**b**–**e**) Densitometric analysis of c-Met (**b**, **c**) and Pten (**d**, **e**) protein levels in surrounding livers (SL) and corresponding HCC (T) as well as in HCC with better (HCCB) and poorer (HCCP) prognosis. HCCB and HCCP are characterized by <3 and >3 years’ survival following partial liver resection, respectively. Optical densities of the peaks were calculated using the Quantity One software (Bio-Rad), normalized to β-actin levels, and expressed in arbitrary units. Student’s *t*-test: ***P<0.0001.

First, we determined the levels of c-Met and Pten in human HCC samples (*n*=50). For this purpose, we examined the protein expression patterns of c-Met and Pten in a collection of human HCC specimens by immunohistochemistry. We found that c-Met levels were upregulated, when compared with non-tumorous surrounding counterparts, in 22 of 50 (44%) of the HCC specimens (Figure 1a). Equivalent levels of c-Met immunoreactivity in HCC and corresponding non-neoplastic livers were detected in the remaining samples (28/50, 56% Figure 1b). As concerns Pten staining patterns, 34 of 50 (68%) HCC showed downregulation when compared with corresponding non-neoplastic counterparts (Figure 1a and b), with no differences in staining intensity between tumorous and non-tumorous tissue in the remaining samples (16/50, 32% not shown). Importantly, the vast majority (16/22, 72.7%) of tumors with upregulation of c-Met belonged to the HCC subclass with shorter survival/poor prognosis (HCCP; as defined by patient’s survival shorter than 3 years following partial liver resection). Downregulation of Pten was also most frequently detected in HCCP (23/34, 67.6%) than in HCC with longer survival/better prognosis (HCCB; patient’s survival longer than 3 years following partial liver resection). Concomitant upregulation of c-Met and downregulation of Pten was detected in 20 HCC specimens, 15 of which belonged to the HCCP subset. Subsequently, we evaluated the protein levels of c-Met and Pten in the HCC sample collection by western blot analysis. We found that c-Met expression was significantly higher in HCC than in corresponding non-tumorous livers (Figure 2a and b). Furthermore, c-Met protein levels were significantly higher in HCCP than in HCCB (Figure 2c). In addition, levels of Pten were significantly lower in HCC when compared to corresponding non-tumorous counterparts (Figure 2a and d), especially HCCP (Figure 2e). No association between the levels of c-Met and Pten and clinicopathologic features of the patients, including age, gender, etiology, presence of cirrhosis, tumor size and tumor differentiation, was detected (data not shown).

Taken together, the present data indicate that upregulation of c-Met and downregulation of Pten often occur in human HCC, especially in the subset associate with poorer prognosis.

### Deletion of Pten synergizes with overexpression of c-Met induces HCC development in mice

**Figure 3:**
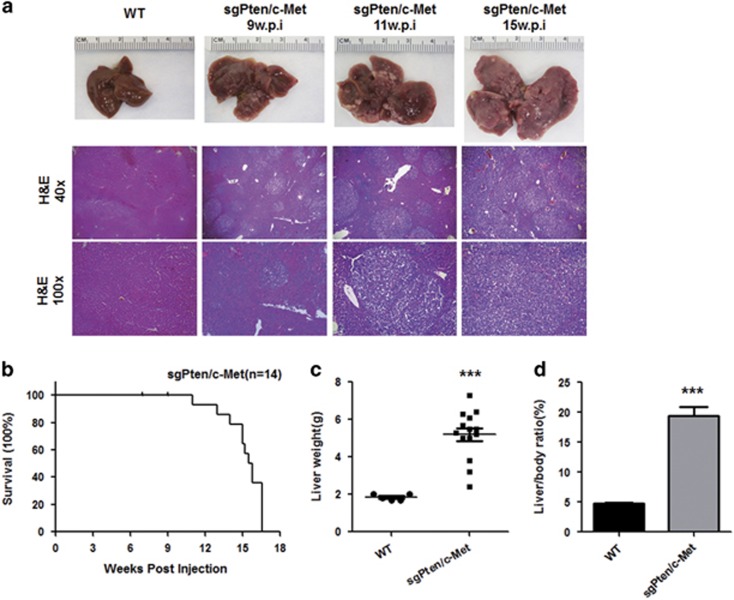
Deletion of Pten synergizes with overexpression of c-Met to induce hepatocellular carcinoma development in mice. (**a**) Gross images and HE staining of wild-type (WT) and sgPten/c-Met mouse livers at indicated time points post hydrodynamic injection. (**b**) Survival curve of sgPten/c-Met-injected mice (*n*=14). (**c**) Liver weight of wild-type (WT, *n*=5) and sgPten/c-Met mice (*n*=14), *P*<0.0001. (**d**) Liver to body weight ratio of wild-type (WT) and sgPten/c-Met mice, *P*<0.0001. Original magnifications in **a**: × 40 (upper panel) and × 100 (lower panel).

Previously, we showed that an activated form of AKT (Myr-AKT) or activated mutant forms of PIK3CA cooperate with c-Met to induce liver cancer.^24, 32^ However, myr-AKT is an artificial construct and PIK3CA mutations are extremely rare in human HCC. In contrast, loss of Pten expression is a frequent genetic event in human HCC (Figure 1).^12, 13^ Furthermore, the present study supports the concomitant loss of Pten and high expression of c-Met in a subset of human HCC specimens (Figures 1 and 2). We therefore hypothesized that deletion of Pten cooperates with c-Met to trigger HCC formation in mice. For this purpose, we applied CRISPR-based gene editing (sgPten) and hydrodynamic injection to delete Pten in the mouse liver.^28^ We found that, consistent with previous findings, overexpression of sgPten alone resulted in the appearance of sporadic hepatocytes with lipid accumulation, but not tumor formation (data not shown).^28^ Long-term overexpression of c-Met alone by hydrodynamically injecting pT3-EF1α-c-Met and pCMV/SB into mice led to liver dysplasia, but not tumor development (not shown), in accordance with previous findings.^33^ Subsequently, we co-injected mice with sgPten, pT3-EF1α-c-Met as well as pCMV/SB (sgPten/c-Met). We found that in sgPten/c-Met mice liver tumors developed as early as ~9 weeks post injection (Figure 3a). By 11–15 weeks post injection, all mice showed abdomen enlargement and were required to be killed (Figure 3b, *n*=14). Upon dissection, numerous tumors could be found throughout the liver, leading to increased liver weight and liver/body ratio (Figure 3c and d).

**Figure 4:**
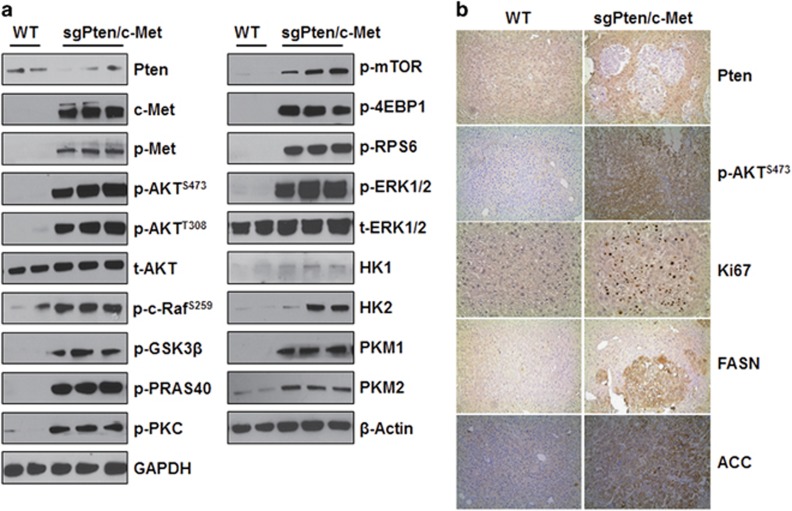
Molecular characterization of hepatocellular carcinomas developed in sgPten/c-Met mice. (**a**) Levels of activation of AKT/mTOR and Ras/MAPK pathways in wild-type (WT) and sgPten/c-Met mouse livers, as detected by western blot analysis. Representative blots are shown. GAPDH and β-actin were used as loading controls. (**b**) Immunohistochemical staining of WT and sgPten/c-Met mouse livers. Original magnifications: × 100 for Pten, p-AKT^S473^, fatty acid synthase (FASN) and acetyl-CoA carboxylase (ACC) proteins; × 200 for Ki67. p, phosphorylated; t, total.

At the histological level, preneoplastic lesions and hepatic adenomas (HCAs) could be found ~9 weeks post injection in the livers of sgPten/c-Met mice. At later time points, liver parenchyma was occupied by confluent HCAs and HCCs (Figure 3a). Tumor lesions were characterized predominantly by a clear-cell phenotype, owing to lipid accumulation, similar to those observed in PIK3CA/c-Met induced liver tumors.^32^ No cholangiocellular lesions were detected in the sgPten/c-Met mouse liver. At the cellular level, loss of Pten expression could be easily visualized in sgPten/c-Met HCC lesions (Figure 4b). Overexpression of c-Met as well as activation of c-Met (p-Met) was validated by western blotting (Figure 4a). Furthermore, sgPten/c-Met tumor cells exhibited consistently higher cell proliferation compared with normal liver (Figure 4b).

At the biochemical level, sgPten/c-Met HCC lesions exhibited high levels of AKT signaling activation, as demonstrated by the increased expression of p-AKT (both S473 and T308) and AKT substrates (p-c-Raf, p-GSK3 and p-PRAS40). Increased p-mTOR, p-PKC, phosphorylated 4E binding protein 1 (p-4EBP1) and ribosomal protein S6 (p-RPS6) were also detected, underscoring the activation of the mTOR cascade in sgPten/c-Met mice. It is well known that aberrant metabolism is one of the hallmarks of cancer; and increased glycolysis and lipogenesis are the major metabolic events downstream of mTOR. Consistently, we found high expression of hexokinase 1 (HK1), hexokinase 2 (HK2) and pyruvate kinase 1 and 2, muscle isoform (PKM1 and PKM2), major enzymes involved in glycolysis, in sgPten/c-Met liver tumor lesions (Figure 4a). Furthermore, high levels of FASN and ACC, key proteins regulating *de novo* lipgenesis, were also detected in sgPten/c-Met tumor cells (Figure 4b). Previous studies have shown that the Ras/MAPK signaling cascade is ubiquitously activated in human HCCs.^34^ Consistently, sgPten/c-Met HCC lesions also displayed increased expression of p-ERK, supporting the activation of Ras/MAPK cascade in these tumor cells.

In summary, our study indicates that loss of Pten synergizes with overexpression of c-Met to induce HCC formation in mice. Tumor cells showed high levels of AKT/mTOR and Ras/MAPK cascades as well as elevated glycolysis and lipogenesis, which are often induced in human HCC samples.

### mTORC2 is required for sgPten/c-Met-driven hepatocarcinogenesis

**Figure 5:**
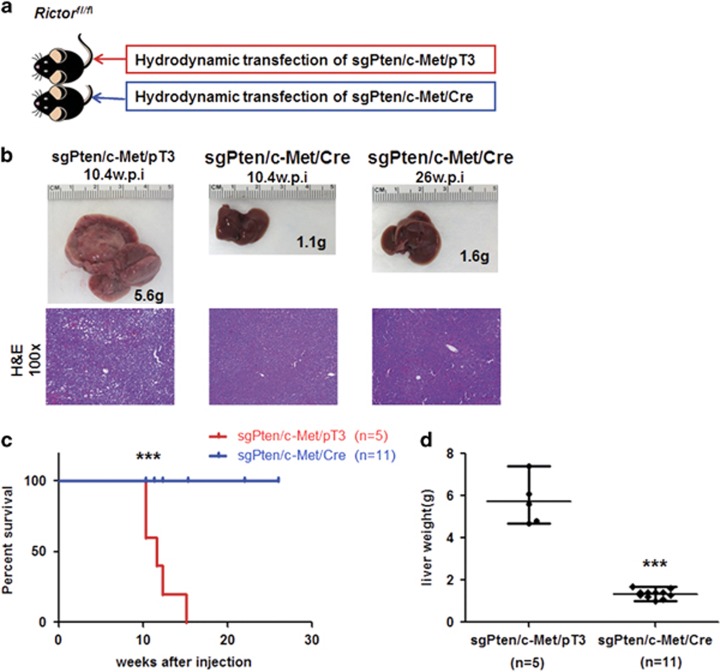
sgPten/c-Met-induced hepatocarcinogenesis is completely abolished by deletion of *Rictor* in mice. (**a**) Scheme of the experiment. (**b**) Gross images and HE staining of sgPten/c-Met/pT3 and sgPten/c-Met/Cre injected *Rictor*^*flox/flox*^ mouse livers at indicated time points. (**c**) Survival curve of sgPten/c-Met/pT3 (*n*=5) and sgPten/c-Met/Cre (*n*=11) injected *Rictor*^*flox/flox*^ mice, *P*=0.0001. (**d**) Liver weight of sgPten/c-Met/pT3 and sgPten/c-Met/Cre injected *Rictor*^*flox/flox*^ mice, *P*<0.0001.

mTORC2 is a key regulator of the AKT signaling in many tumor entities, including HCC.^19, 35^ Increased p-AKT expression in sgPten/c-Met HCC lesions supports the activation of mTORC2 in tumor cells. Thus, sgPten/c-Met mice provide us with an excellent *in vivo* model to investigate the functional role of mTORC2 in hepatocarcinogenesis. For this purpose, we inactivated the mTORC2 pathway using conditional *Rictor* knockout mice (*Rictor*^*fl/fl*^ mice).^29^ Specifically, we hydrodynamically injected sgPten, c-Met and Cre plasmids into *Rictor*^*fl/fl*^ mice (sgPten/c-Met/Cre; *n*=11), thus allowing the simultaneous expression of sgPten and c-Met oncogenes, while deleting *Rictor* in a subset of mouse hepatocytes. As a control, sgPten, c-Met and pT3-EF1α (empty vector) were co-injected into *Rictor*^fl/fl^ mice (sgPten/c-Met/pT3, *n*=5) (Figure 5a).

**Figure 6:**
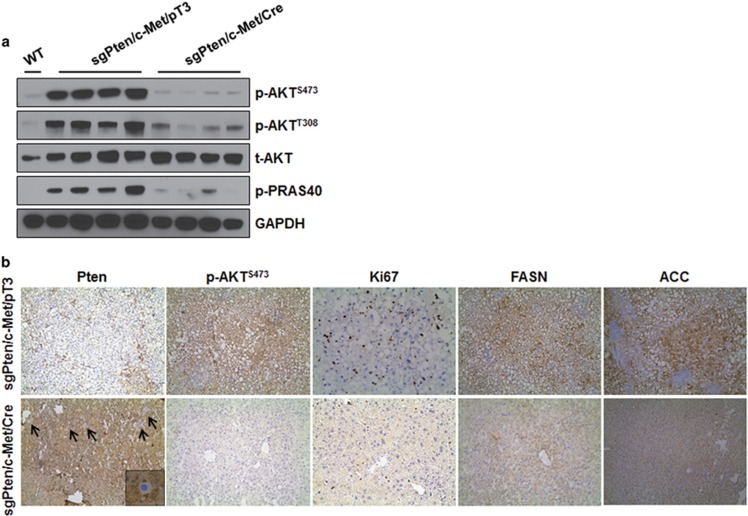
Molecular characterization of hepatocellular carcinoma developed in sgPten/c-Met *Rictor*^*flox/flox*^ mice. (**a**) Representative immunoblotting in wild-type (WT), sgPten/c-Met/pT3 and sgPten/c-Met/Cre *Rictor*^*flox/flox*^ mouse liver tissues. GAPDH was used as loading control. (**b**) Immunohistochemical staining. Arrows indicate scattered Pten(−) hepatocytes in the liver. The inset shows a single hepatocyte that has lost Pten immunoreactivity. Original magnifications: × 100 for Pten, p-AKT^S473^, fatty acid synthase (FASN) and acetyl-CoA carboxylase (ACC)s; × 200 for Ki67. p, phosphorylated; t, total.

Noticeably, all sgPten/c-Met/pT3 mice developed liver tumors by 10–15 weeks post injection (Figure 5b), whereas no macroscopic or histopathological alterations were detected in the livers of sgPten/c-Met/Cre mice up to 26 weeks post hydrodynamic injection (Figure 5c and d; Table 1). Histological evaluation showed that HCA and HCC lesions occupied most of the liver parenchyma in sgPten/c-Met/pT3 injected *Rictor*^*fl/fl*^ mice, similar to that observed in sgPten/c-Met injected FVB/N mice (Figure 5b). Importantly, as the tumors derived from individually transformed hepatocytes that lost Pten protein expression, tumor cells were found to be Pten (−) (Figure 6b). Loss of Pten protein led to the increased expression of p-AKT, its downstream effector p-PRAS40, as well as high expression of FASN and ACC (Figure 6a and b). In contrast, liver from sgPten/c-Met/Cre injected *Rictor*^*fl/fl*^ mice appear to be completely normal, indistinguishable from un-injected mouse liver (Figure 5b). In the liver tissues from sgPten/c-Met/Cre-injected *Rictor*^*fl/fl*^ mice, only a few hepatocytes successfully underwent CRISPR mediated gene editing, leading to the loss of Pten protein expression. However, these Pten(−) hepatocytes failed to transform into tumor cells; thus, they remained as normal hepatocytes and did not expand. Indeed, individual Pten(−) hepatocytes could be readily found scattered throughout the liver in sgPten/c-Met/Cre *Rictor*^*fl/fl*^ mice (Figure 6b). Consistently, only low levels of p-AKT, p-PRAS40, FASN and ACC expression were observed in liver tissues from sgPten/c-Met/Cre *Rictor*^*fl/fl*^ mice (Figure 6a and b).

In summary, our data suggest that an intact mTORC2 signaling is required for sgPten/c-Met-induced liver carcinogenesis, and provide strong evidence to support the critical role of mTORC2 during liver tumor development.

## Discussion

The mTOR cascade is a major signaling pathway regulating cell growth and metabolism. Previous studies have demonstrated the critical role of mTOR in HCC.^13^ In particular, it has been shown that components of mTORC1 and mTORC2, including p-RPS6, p-AKT and Rictor, are upregulated in ~50% of HCC specimens.^13, 19^ In addition, activation of mTOR has been associated with poor prognosis and early recurrence independent of the underlying etiology of HCC.^36^ Recently genomic studies demonstrate that mutations in the mTOR pathway, including mTOR and PIK3CA are rather rare in liver cancer. In this tumor type, deregulation of EGF and Pten pathways has been rather associated with aberrant mTOR activation.^13, 37^ In human HCC, Pten can be downregulated via multiple mechanisms, including promoter methylation, post-transcriptional targeting by miR-21, post-translational modulation by the NEDD4-1 ubiquitin ligase and so on.^37^ The critical role of loss of Pten in regulating mTOR pathway and promoting liver tumorigenesis has been established using liver specific *Pten* knockout mice.^38, 39^ Indeed, it has been found that 100% of *AlbCre;Pten*^*fl;fl*^ mice developed liver tumors by 12 months of age. These tumors consisted of mixed HCC and cholangiocarcinomas (CCA).^39^ As mixed HCC and CCA tumors are rather rare entities in humans, it was mandatory to generate a pure HCC model depleted of Pten to further study the molecular signaling triggered by Pten loss in hepatocarcinogenesis.

Hydrodynamic transfection, which combines hydrodynamic tail vein injection with SB-mediated somatic integration, has become an increasingly popular method for *in vivo* modeling of liver tumorigenesis.^30^ However, the approach is especially successful in inducing liver tumors by the overexpression of oncogenes.^30^ In order to generate liver tumors with deletion of a given tumor suppressor gene using hydrodynamic transfection, the only feasible approach is to apply shRNA-based gene silencing. However, the shRNA sequence is highly toxic to hepatocytes (Wang C, unpublished data), and only miR-30-based shRNA can be used *in vivo*.^40^ Furthermore, shRNA leads to partial gene silencing, not complete knockout of the target gene. Thus, shRNA-based gene silencing may not be sufficient to induce a phenotype. CRISPR-mediated gene editing is a powerful technology that allows the complete elimination of a target gene in cells.^41, 42^ Recently, Xue *et al.*^28^ demonstrated that combining CRISPR-mediated gene silencing and hydrodynamic injection may be a reliable and efficient way to knockout a target gene in the liver. Specifically, the study showed that hydrodynamic injection of plasmids encoding CRISPR-based gene knockout for Pten and TP53 (sgPten and sgTP53) led to the formation of CCA in mice.^28^ In the present study, we found that CRISPR-based gene knockout of a tumor suppressor can be combined with SB-mediated somatic integration of an oncogene to induce liver tumor development *in vivo*. Specifically, we demonstrated that loss of Pten (using sgPten) synergized with overexpression of c-Met to promote HCC formation. The resulting tumors showed loss of Pten and high expression of c-Met, as well as their downstream effectors, including activation of the AKT/mTOR and Ras/MAPK cascades. Thus, our present investigation supports the combination of hydrodynamic transfection with CRISPR mediated gene editing for modeling liver tumor *in vivo*. It is important to note that unlike liver specific *Pten* knockout mice, which develop both HCC and CCA, only HCC lesions (but no CCA) were found in sgPten/c-Met mice. Therefore, sgPten/c-Met mice represent an excellent model to study the consequences of Pten loss during HCC development.

While mTORC1 has been extensively studied in tumorigenesis, including HCC, the functional contribution of mTORC2 in cancer initiation and progression remains poorly characterized.^43^ In HCC, the investigations on mTORC2 are still very limited. Activated AKT is found in ~50% of human HCCs,^21^ and AKT activation requires an intact mTORC2.^23^ In addition, previous studies showed that chromosomal gains at Rictor locus and Rictor overexpression are present in a subset of human HCC samples and associated with early tumor recurrence.^13, 23^ Interestingly, our most recent analysis using the TCGA data set suggests that mSin1, another unique mTORC2 component, is upregulated in human HCC samples (Che L, unpublished data), further supporting the role of mTORC2 in human liver cancer. Obviously, further experiments are required to elucidate the mechanisms whereby mTORC2 is activated in human HCC. In this study, using sgPten/c-Met mouse HCC model in combination with *Rictor* conditional KO mice, we demonstrate that mTORC2 is required for sgPten/c-Met-driven hepatocarcinogenesis. The results provide solid *in vivo* evidence for the important role of mTORC2 along hepatocarcinogenesis. Mechanistically, we showed that loss of mTORC2 inhibited AKT activation induced by sgPten/c-Met co-expression. mTORC2 regulates multiple AGC kinases; among them, AKT proteins are considered to be the major mediators.^20^ In the liver, AKT1 and AKT2 are the only isoforms expressed. Studies have shown that both AKT1 and AKT2 contribute to liver regeneration after partial hepatectomy,^44^ and may play distinct roles in liver tumorigenesis.^45^ Further experiments are necessary to identify the kinases downstream of mTORC2 that are required for sgPten/c-Met induced HCC formation in mice.

HCC is a lethal malignancy worldwide, and a better understanding of the signaling pathways involved in its molecular pathogenesis is required for the development of effective treatments. Conventional chemotherapy has proven to be ineffective to treat HCC patients. Thus, the development of novel targeted therapies is the alternative approach. Mouse models are critical tools to evaluate the therapeutic potential of these new drugs. As mTOR cascade has been shown to be a key regulator of tumorigenesis, mTOR inhibitors have been developed and tested clinically in various tumor types, including HCC.^46^ It is important to note that the first generation of mTOR inhibitors, the Rapamycin homologs (Rapalogs), including Everolimus, are able only to partially inhibit mTOR. Specifically, Everolimus inhibits the S6K/RPS6 axis downstream of mTORC1, but it does not affect the 4EBP1/eIF4E branch of mTORC1 nor it inhibits mTORC2. As a consequence, it is not surprising that clinical studies demonstrated very limited efficacy of Everolimus for cancer treatment. In HCC, Everolimus treatment failed to improve overall patients’ survival.^47^ The second generation mTOR inhibitors include pan-mTOR and PI3K/mTOR inhibitors. These new inhibitors can suppress both mTORC1 and mTORC2, leading to increased anti-tumor efficacy. Currently, there are only limited studies on the efficacy of second generation mTOR inhibitors for HCC treatment.^48^ Importantly, the sgPten/c-Met murine HCC model shows high levels of mTORC1 and mTORC2 activities, and our investigation demonstrates its dependence by mTORC2. Therefore, sgPten/c-Met mice are an excellent preclinical model for testing the therapeutic efficacy of the new class of mTOR inhibitors in HCC.

## Figures and Tables

**Table 1:** Detailed mouse data from sgPten/c-Met/pT3 or sgPten/c-Met/Cre-injected *Rictor* ^*fl/fl*^ mice

*Injection*	*Gender*	*Weeks post injection*	*Body weight (g)*	*Liver weight (g)*	*Histology*
sgPten/c-Met/pT3	F	10.4	24.6	6.1	HCC
	F	10.4	22.1	5.6	HCC
	M	11.7	27.7	4.7	HCC
	M	12.4	29.4	7.4	HCC
	F	15.1	22.7	4.8	HCC
					
sgPten/c-Met/Cre	F	10.4	23.4	1.1	Normal liver
	F	10.4	23.1	1.0	Normal liver
	M	11.3	32.0	1.4	Normal liver
	M	11.3	33.5	1.4	Normal liver
	M	12.3	30	1.3	Normal liver
	M	15.3	31	1.3	Normal liver
	M	15.3	32.8	1.2	Normal liver
	M	22	34.8	1.4	Normal liver
	M	26	33.2	1.4	Normal liver
	M	26	37.3	1.6	Normal liver
	M	26	40.4	1.7	Normal liver
