# Supplementary material for: Loss of Pten synergizes with c-Met to promote hepatocellular carcinoma development via mTORC2 pathway
Source: Exp Mol Med. 2018 Jan 5;50(1):e417–. doi: 10.1038/emm.2017.158 (PMC5992985; doi:10.1038/emm.2017.158)
Supplement: Supplementary Table 1 [file emm2017158x1.doc]

**Supplementary Table 1**. Clinicopathological features of HCC Patients

| Variables Features | | |
| --- | --- | --- |
|  | HCCB^a^ | HCCP^b^ |
| No. of patients  Male  Female | 25  20  5 | 25  19  6 |
| Age (Mean ± SD) | 60.2  ± 12.2 | 64.5  ± 14.0 |
| Etiology  HBV  HCV  Ethanol  Wilson’s disease  Hemochromatosis | 11  8  4  1  1 | 11  9  4  1  0 |
| Cirrhosis  +  - | 19  6 | 20  5 |
| Tumor size  > 5 cm  < 5 cm | 15  10 | 15  10 |
| Edmondson and Steiner grade  II  III  IV | 8  10  7 | 9  10  6 |
| Alpha-fetoprotein secretion  > 300 ng/ml of serum  < 300 ng/ml of serum | 17  8 | 15  10 |
| Survival after partial liver resection (months)  Means ± SD | 56.5  ± 17.8 | 17.1  ± 9.25 |

^a^HCCb, HCC with better prognosis/longer survival (survival >3 years following partial liver resection)

^b^HCCp, HCC with poorer prognosis/shorter survival (survival <3 years following partial liver resection)
